# Exploring the psychosexual impact and disclosure experiences of women testing positive for high‐risk cervical human papillomavirus

**DOI:** 10.1111/bjhp.12612

**Published:** 2022-07-12

**Authors:** Kirsty F. Bennett, Jo Waller, Julia V. Bailey, Laura A. V. Marlow

**Affiliations:** ^1^ Cancer Communication and Screening Group, Department of Behavioural Science and Health University College London London UK; ^2^ Cancer Prevention Group, School of Cancer and Pharmaceutical Sciences King's College London London UK; ^3^ e‐Health Unit, Department of Primary Care and Population Health University College London London UK

**Keywords:** cancer, cervical cancer, cervical screening, disclosure, human papillomavirus, psychological, psychosexual, psychosocial, qualitative research

## Abstract

**Objectives:**

To examine the psychosexual impact and disclosure experiences of women testing HPV‐positive following cervical screening.

**Design:**

In‐depth semi‐structured interviews.

**Methods:**

Interviews were conducted with 21 women of screening age (i.e. those aged 24–65 years) in England who self‐reported testing HPV‐positive in the context of cervical screening in the last 12 months. Data were analysed using Framework Analysis.

**Results:**

The sexually transmitted nature of HPV, and aspects relating to the transmission of HPV and where their HPV infection had come from, had an impact on women's current, past and future interpersonal and sexual relationships. Most women had disclosed their HPV infection to others, however the factors influencing their decision, and others' reactions to disclosure differed. The magnitude and extent of psychosexual impact was influenced by how women conceptualized HPV, their understanding of key aspects of the virus, concerns about transmitting HPV and having a persistent HPV infection.

**Conclusions:**

Increasing knowledge of key aspects of HPV, such as its high prevalence and spontaneous clearance, and the differences between HPV and other STIs, may increase women's understanding of their screening result and reduce any negative psychosexual consequences of testing HPV‐positive. Referring to HPV as an infection that is passed on by skin‐to‐skin contact during sexual activity, rather than an STI, may help to lessen any psychosexual impact triggered by the STI label.

**Statement of Contribution**

**What is already known on this subject?**

- Quantitative research suggests that psychosexual distress is elevated among women testing HPV‐positive.
- Psychosexual concerns include where an HPV infection came from and the potential to transmit HPV to a sexual partner.
- Some women have concerns about disclosing HPV to a sexual partner, partly because of the stigma associated with having an STI and uncertainty about how their partner would respond.

**What does this study add?**

- This study is the first to explore psychosexual and disclosure‐related outcomes in‐depth among women testing HPV‐positive.
- The sexually transmitted nature of HPV impacted women’s current, past and future interpersonal and sexual relationships.
- Women’s knowledge and conceptualisation of HPV influenced their response to testing positive.

## INTRODUCTION

Infection with high‐risk human papillomavirus (HPV) is the cause of virtually all cases of cervical cancer (Bosch et al., 1995; Bosch et al., 2002; Walboomers et al., 1999). HPV is a common sexually transmitted infection (STI) transmitted through skin‐to‐skin genital contact during sexual activity. Around 80% of individuals will acquire a genital HPV infection by age 50, however most infections are asymptomatic and clear spontaneously within 2 years (Franco et al., 1999; Giuliano et al., 2002; Koutsky et al., 1988; Plummer et al., 2007; Satterwhite et al., 2013; Winer et al., 2011).

Cervical screening programmes in several countries, including England, have introduced HPV primary screening because of its higher sensitivity for detecting high‐grade cervical abnormalities compared to cytology‐based screening (Cuzick et al., 2006; Ronco et al., 2010; Ronco et al., 2014). Individuals attending cervical screening are told that they are HPV‐negative or HPV‐positive, with cytology used to triage HPV‐positive results. In England, individuals testing HPV‐positive with normal cytology are rescreened 12 months' later to see if their HPV infection has cleared, and those testing HPV‐positive with abnormal cytology are referred to colposcopy. In the HPV primary screening pilot in England, 13% received an HPV‐positive result (Rebolj et al., 2019).

HPV primary screening means that some individuals will be told they have an STI following screening. This has potential psychosexual consequences. Psychosexual functioning includes feelings, worries and concerns that relate to, or impact on, sexual behaviour or sexual relationships (Bennett, Waller, McBride, et al., 2021a).

Quantitative research suggests that psychosexual distress is elevated among women testing HPV‐positive (Bennett, Waller, McBride, et al., 2021a). A systematic review of 25 studies identified a range of psychosexual concerns in the qualitative literature following an HPV‐positive result (Bennett et al., 2019). Common concerns related to source of the infection and the potential to transmit HPV to a sexual partner. Another review (Bennett, Waller, Ryan, et al., 2021b) found that some women have concerns about disclosing HPV to a sexual partner, partly because of the stigma associated with having an STI and uncertainty about how their partner would respond. The studies included in these reviews were all carried out prior to 2018. It is likely that awareness and understanding of HPV has changed since the introduction of HPV primary screening. In addition, while previous studies have highlighted psychosexual and disclosure‐related outcomes, no study has explored these issues in depth. There is some evidence that psychosexual distress may be greater among women who are not in a relationship (Hsu et al., 2018). Research suggests that there are differences in anxiety (McBride et al., 2020), and psychosexual impact (Bennett, Waller, McBride, et al., 2021a) by cytology result.

The aim of this study was to qualitatively explore the psychosexual impact and disclosure experiences of women who had tested HPV‐positive in the context of HPV‐based cervical screening. To our knowledge, this is the first study to explore the psychosexual impact and disclosure experiences of women testing HPV‐positive since the introduction of HPV primary screening.

## METHODS

### Study design

In‐depth interviews were conducted with women of screening age in England (i.e. those aged 24–65 years) who self‐reported having tested HPV‐positive (with normal or abnormal cytology) in the context of cervical screening in the last 12 months. Women were eligible to take part in the study if they spoke English and were able to give informed consent.

### Ethics

Ethical approval was obtained from University College London's Research Ethics Committee (6930/003).

### Participants

Participants were predominantly recruited through Saros (https://www.sarosresearch.com/), a market research recruitment agency with a database of over 300,000 participants. All women aged 24 to 65 years and living in England were invited by email to take part in the study (n = 37,159). To assess eligibility for the study and enable recruitment of a range of women with different characteristics, women expressing an interest in the study completed a questionnaire assessing age, relationship status, ethnicity, education level, cytology result and HPV knowledge. In addition, two items from the HPV Impact Profile assessed sexual impact following their most recent cervical screening result (Mast et al., 2009). In total, 5% of women who expressed an interest met the study's eligibility criteria (n = 89). Women were purposively sampled to include those with normal and abnormal cytology results and those who were/were not in a relationship.

An advert for the study was also placed on the Jo's Cervical Cancer Trust ‘Take part in new research’ webpage. The advert included a brief description of the study and the first authors contact details for women who were interested in taking part in the study or those who wanted more information. The study was advertised from September 2019 to July 2020. During this period, three women expressed an interest in taking part in the study and one participant was recruited to the study.

### Procedure

Interviews were carried out by the first author at a convenient time for the participant and took place over the phone or via video call in June and July 2020. A topic guide was used to guide the interviews (see Appendix S1). Interviews were audio‐recorded and transcribed verbatim. Participants received a £40 voucher as a thank you for taking part.

### Analysis

Data were analysed using Framework Analysis, a ‘matrix‐based method for ordering and synthesising data’ to analyse qualitative data (Ritchie & Lewis, 2010). Framework Analysis aims to identify similarities and differences, and subsequently look for relationships in qualitative data, with the aim of generating descriptive and/or explanatory conclusions centred around themes (Gale et al., 2013).

After familiarization with the data, recurring themes or ideas were identified and a working thematic framework or ‘index’ of recurrent themes developed. Themes were sorted and grouped into a smaller number of higher order categories or main themes. The thematic framework was then applied to the data.

Thematic charts were constructed using the thematic framework and data from the transcripts were synthesized and placed in the thematic charts. Each theme was displayed in a separate chart, with columns representing subthemes and each row representing a participant. The development of the thematic framework and synthesis of data into the thematic charts were carried out by KB and reviewed and discussed with LM. The thematic framework was an iterative process until the framework was considered appropriate for the data.

Data were analysed by KB. During the interpretation phase of the analysis, KB presented her interpretations to all authors on several occasions and the findings were discussed. The interpretation phase was an iterative process.

Data were analysed in NVivo 12 PRO and stored and managed in Microsoft Excel.

## RESULTS

Interviews were carried out with 21 women (mean age: 39.8 years; range 25–64) and lasted from 21–70 minutes. Participants included women who were in a relationship (*n* = 10), single (*n* = 10) and one woman who was dating/in a casual relationship. Women reported that they were HPV‐positive with normal cytology (*n* = 10) or HPV‐positive with abnormal cytology (*n* = 11). Participant characteristics are shown in Table 1.

**TABLE 1:** Participant characteristics

	Number
Age	
25–34	7
35–44	8
45–54	5
55–65	1
Self‐reported cytology result	
Normal	10
Abnormal	11
Relationship status	
In a relationship^a^	10
Not in a relationship (i.e. single)	10
Dating/in a casual relationship	1
Ethnicity	
White (British or other)	14
Asian	3
Black	1
Mixed/multiple	3
Education	
Master's degree or higher	5
Degree	8
A Levels	5
GCSEs	3
Self‐reported HPV knowledge	
Very good	‐
Good	5
Fair	9
Poor	5
Very poor	2
First HPV result^b^	
Yes	14
No	7
Having less sex (mean score [standard deviation])^c^	5.66 (3.62)
Satisfaction with sex life (mean score [standard deviation])^c^	3.95 (2.77)

^a^ This included women who were in a relationship, living with a partner, married or in a civil partnership.

^b^ Women were asked during the interview whether this was their first HPV‐positive result.

^c^ Scale range: 0–10, where 0 indicates ‘Not at all’ and 10 indicates ‘Extremely’.

**FIGURE 1:**
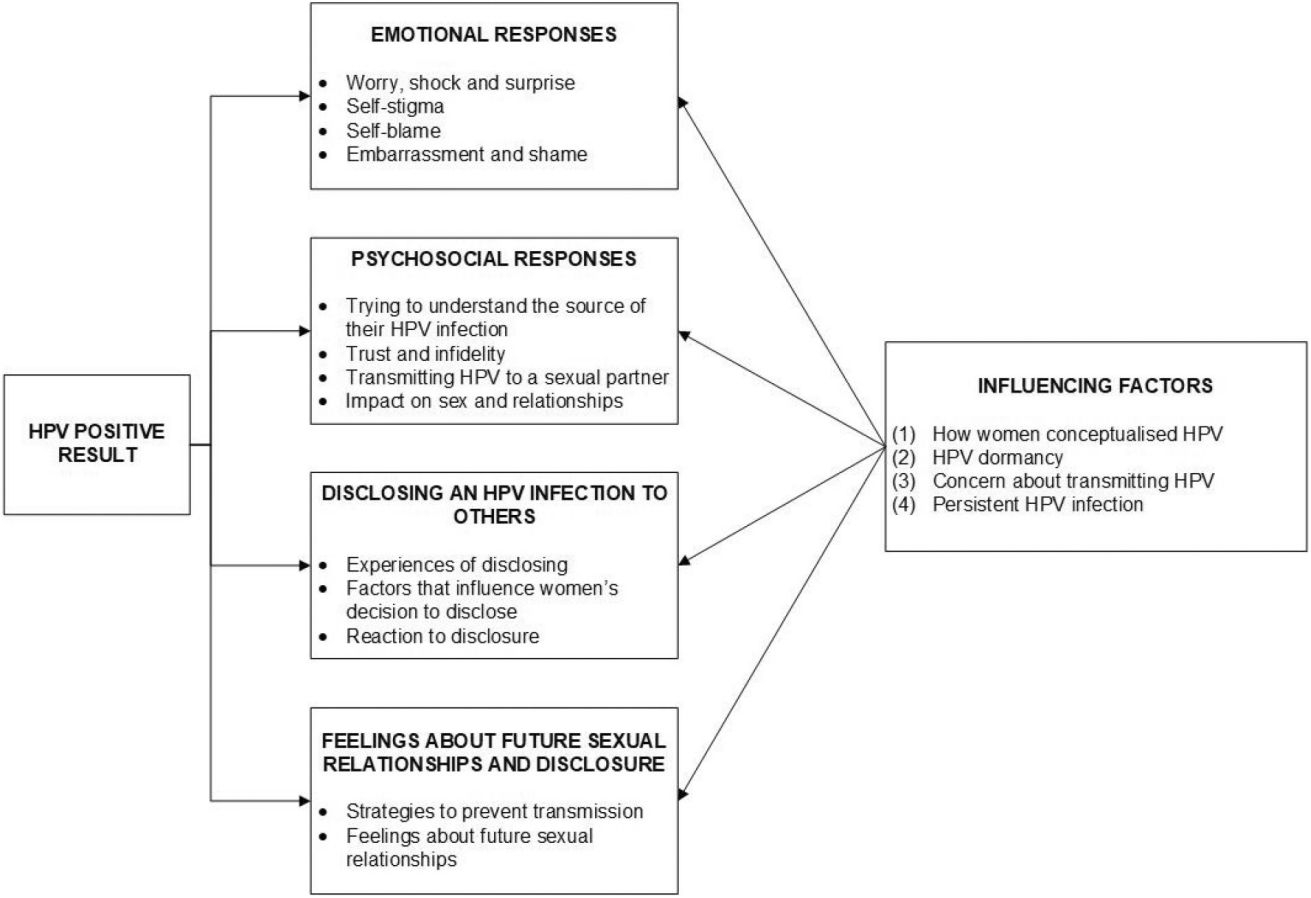
A model of psychosexual responses to an HPV‐positive result

Women's responses to testing HPV‐positive are summarized in Figure 1. The main topics discussed by women fitted into four domains: (1) Emotional responses,   (2) Psychosocial responses, (3) Disclosing an HPV infection to others and, (4) Feelings about future sexual relationships and disclosure. Several factors appeared to influence women's emotional and psychosocial responses and minimize the potential negative impact of testing HPV‐positive: (1) How women conceptualized HPV, (2) HPV dormancy, (3) Concern about transmitting HPV and, (4) Persistent HPV infection. Women's responses and the influencing factors are described in the following sections with example quotes.

### Emotional responses

#### Worry, shock and surprise

Women felt worried and concerned when they received their HPV‐positive result, because they did not know what HPV was or were concerned about developing cancer. Some felt shocked, surprised or panicked at having an STI because they had been with their current partner for several years, or had not had unprotected sex and questioned whether their result was correct because they were not ‘that sort of person’.

…I was absolutely panic‐stricken because I thought, “Oh my God, this sounds awful. I've got a sexually transmitted disease at my age. Where have I got this from?” You know. Ohhh! (P15, 35–44, normal cytology, not in a relationship) ^1^

I was a bit shocked cos I thought, “Well, I'm with the same person that I've been with for like a long time”. (P19, 45–54, abnormal cytology, in a relationship)

#### Self‐blame

Women blamed themselves for acquiring HPV because they had had unprotected sex or had several sexual partners around the same time, or felt that potential future issues regarding fertility or pregnancy could be viewed as their fault:

I think it's just because you just hear so many things, don't you, of unprotected sex and you know you shouldn't do it, but I did (P8, 35–44, abnormal cytology, in a relationship).

With future pregnancy…I think it makes you worry that everything will be alright, and then it kind of puts this weight on you if it doesn't go, you feel that it could be, you know, your fault somehow (P1, 25–34, abnormal cytology, in a relationship).

#### Self‐stigma, embarrassment and shame

Women described feeling ‘dirty’, ‘horrible’, ‘grim, and infected and nasty’ when they received their result and commented on the stigma or negative connotation that is associated with ‘…anything that's sort of a bit sexually orientated’.

‘I felt a bit dirty with it, like it felt like I did have some kind of infection’ (P23, 25–34, abnormal cytology, not in a relationship).

Embarrassment and shame at having something acquired through sexual activity was mentioned, often where women conceptualized HPV as an STI or something they had got through unprotected sex. Feeling ashamed was described because they associated STIs with promiscuity, or felt that information provided about HPV implied this, and some questioned how they had got HPV when they had not had ‘loads of partners’ and ‘didn't sleep around’. The stereotype of someone who gets HPV differed from women's view of themselves which made them feel uncomfortable:

…this stereotype of someone that get, that gets HPV, people that have unprotected sex and lots of partners. And because that wasn't the case with me I think that brought up the shame. It's like oh, they probably think I'm promiscuous or… Do you know what I mean? So it was definitely uncomfortable (P9, 35–44, normal cytology, not in a relationship).

Receiving an HPV‐positive result felt different from receiving an abnormal cytology result due to its sexually transmitted nature and the stigma associated with this:

…yeah, ‘cause that's different, it takes away that kind of like dirty, shameful stigma of it I guess, if you know, knowing that it's not something that's kind of been caught or picked up. It was just a change in cells, it was, yeah. You can associate that differently I guess (P23, 25–34, abnormal cytology, not in a relationship).

Embarrassment and shame were mentioned by women regardless of their relationship status.

### Psychosocial responses

#### Trying to understand the source of their HPV infection

A common response was to try to understand where an HPV infection had come from. Women in long‐term relationships were particularly confused about this. Some assumed that the infection had come from a current or recent sexual partner because previous cervical screening test results had been normal, or HPV had not been found:

…I'm now thinking like well who did I get it from, who did I, who did I pick it up from. It must have been, you know. I'm, in my head I'm thinking it must have been somebody recently because otherwise it would have been picked up on my first two smears (P23, 25–34, abnormal cytology, not in a relationship).

Others felt unable to determine which sexual partner had given them HPV. Women not in a relationship questioned how they had got HPV as they had not recently been sexually active:

I didn't know anything about it and I was quite intrigued… not intrigued by it, but I was like, oh, I wonder why I've got it, you know, or how because at that point I was like… I'd not had sex for a few years, couple of years (P21, 35–44, abnormal cytology, dating/in a casual relationship).

#### Trust and infidelity

Women's attempts to understand the source of their HPV infection led to issues around trust of their partner, particularly among women who lacked knowledge about HPV, and this had the potential to have an impact on their relationship:

Um with the previous relationship um when I first had the screening and the results, it definitely did have an impact and I think that… I suppose quite young at that point and because of the lack of understanding of it, you just… you are not sure. I suppose trust plays a part in it and it makes like your partner reflect on your history and how did you get it and um did I give it or did you have it before, those sorts of questions… (P4, 25–34 years, abnormal cytology, in a relationship).

Some women trusted their partner and did not suspect any infidelity, however others raised concerns that their partner had been unfaithful and questioned whether that was how they had acquired HPV:

…my immediate reaction to him was, “Well, what have you been up to?” [laughter] and I just thought, “Well, you've got it off somebody. It's not off me”…I was a bit like… you know, “have you been cheating on me?” [laughter]. (P15, 35–44, normal cytology, not in a relationship)

Questioning partners about whether they were the source of an HPV infection had an impact on relationships because partners were unhappy about being accused of infidelity, which led to tension or arguments. For one woman, perceiving her partner to be the source of her HPV infection was having an ongoing impact on her relationship.

#### Transmitting HPV to a sexual partner

There was uncertainty around whether it was possible to transmit HPV to a male sexual partner. Women reported feeling ‘guilty’ and worried about potentially having transmitted HPV and were concerned that there was no way of their partner knowing if they had HPV. Concern about transmitting HPV led one woman to question whether she was ‘allowed’ to have sex:

…I said to the nurse “oh am I still allowed to have sex whilst I've got this?” and she was like “yeah of course you are” and then I was like but am I gonna pass it on to someone, you know. So, erm, yeah, that, that would be my biggest concern (P23, 25–34, abnormal cytology, not in a relationship).

Concerns about HPV harming or having an impact on their partners' health were predominantly mentioned by women who had only received one HPV‐positive result. A lack of understanding about the possible impact of HPV resulted in partners being hesitant to have sex. Concern about transmitting HPV to a sexual partner and them passing HPV onto someone else in the future resulting in a ‘chain of people that could be affected’ was mentioned:

…I wouldn't like to think that I did and then if we broke up, they pass it on to someone else. I wouldn't like to think that's sort of… you know, it was a never‐ending cycle and someone else could be in the position that I'm in in the future (P21, 35–44, abnormal, dating or in a casual relationship).

Whether it was possible to be reinfected with HPV, either by their current, or a future sexual partner was queried, with condoms being used to prevent reinfection. Concerns about transmitting HPV to a sexual partner were mentioned by both women in a relationship and those not in a relationship.

#### Impact on sex and sexual relationships

Having HPV had an impact on some women's sexual relationships or feelings or attitudes towards sex. Reduced interest in and frequency of sex were the changes most often described. Some stopped having sex completely because of concerns about transmitting HPV:

Well, we haven't had sex since. So that's a long time…Um, I think he is… I, I would, personally, um, but my boyfriend is unsure. He doesn't wanna catch anything and then resent me for it (P12, 35–44, normal cytology, in a relationship).

Self‐stigma also had an impact on women's interest in sex, affecting their confidence, self‐esteem and sexual self‐image:

So, for the next few weeks, I really did avoid sex, erm because I just felt grim, and infected and nasty (P17, 35–44, normal cytology, in a relationship).

Reduced interest in having sex with someone who they perceived was the source of their HPV infection was described and this led, in one case, to a relationship ending:

…I was seeing someone at the time and I just kind of assumed that they had given it to me. Erm, and it probably actually ended our relationship… I had no interest like in that, like in having sex with him again (P23, 25–34, abnormal cytology, not in a relationship).

Concerns that a partner might end a relationship because of reduced frequency of sex less was also mentioned.

Other concerns about having sex included making abnormal cells or an HPV infection worse. Experiencing pain or discomfort during or after sex was mentioned, however it was felt that the impact this had on sexual pleasure was due to physical symptoms. HPV had a positive impact on one woman's relationship, improving communication and making her and her partner appreciate each other more.

The impact of HPV on sex and sexual relationships was only mentioned by women who were in a relationship.

### Disclosing an HPV infection to others

Most women had disclosed their HPV‐positive result to a partner, friends or family. Some women who were HPV‐positive with abnormal cytology chose to focus on their abnormal cytology result because of the stigma associated with having an STI.

#### Experiences of disclosing

Disclosure was described as ‘awkward’, ‘hard work’ and explaining the circumstances surrounding acquiring HPV ‘embarrassing’ because of the sexually transmitted nature of HPV. Some women gave their partner their results letter to read because they found the letter informative or reassuring. The high prevalence of HPV was mentioned during disclosure. Disclosure was a ‘nerve‐racking experience’ as women were unsure how to approach disclosure and how their partner would respond and were concerned that might want to ‘walk away’ from their relationship or question how they got HPV:

‘I kind of expected my partner to say to me, “Who do you think you caught this from?” and to want to backtrack through my sexual history’ (P17, 35–44, normal cytology, in a relationship).

#### Factors that influence women's decision about whether to disclose HPV

Several factors appeared to influence women's decision about whether to disclose their HPV infection to a sexual partner. Sharing health‐related information was considered normal practice for some. In contrast, others felt cervical screening was infrequently discussed and having HPV had not ‘come up’. Some women did not want others to worry about their HPV‐positive result or were concerned about the impact of disclosure on their relationship:

…I certainly didn't want to cause erm anxiety in the relationship…So, I thought maybe on balance, I decided not to tell him. And he still doesn't know now… As it's something that your body can clear itself of quite quickly, or over a period of, you know, and it doesn't cause any symptoms, I didn't want him to think maybe I'd been having an affair [laughs] with someone or anything, which I wasn't, erm, but I just didn't want to create that atmosphere (P13, 45–54, normal cytology, not in a relationship).

One woman felt she would ‘probably’ tell future sexual partners but had not told her current partner.

Some believed that HPV would not adversely affect their partner so there ‘isn't really any need to discuss it’. Uncertainty about whether it was possible to transmit HPV and the potential impact for men led to questions about whether disclosure was necessary. Conflicting information about disclosure online resulted in women feeling ‘quite confused and concerned about that area of my life’. Being told that HPV was sexually transmitted but receiving guidance with their results informing them that they did not need to tell their partner was ‘confusing and upsetting’.

Although concerns about disclosure were expressed, worry about transmission or feeling that it was the right and thing to do, regardless of the guidance they had received with their results, sometimes resulted in disclosure:

‘Erm, the letter where it said “You don't have to tell your partner,” when I read that line, I immediately thought “I would never do that, I would never not tell someone, they have a right to know.” So, instantly I thought I should tell him. I never thought, I never considered not telling him’ (P17, 35–44, normal cytology, in a relationship).

#### Reaction to disclosure

There was little or no reaction from some women's partners when they disclosed their HPV infection, with women describing partners as being ‘pretty laid back about it all’:

‘I was like, “You haven't had any symptoms, you probably won't, but obviously keep an eye on it.” Erm and that was that really. He wasn't too bothered either because I think he was just the same as me; the people, the right people know about it, and if there's an issue, they'll find it at the next screening…’ (P16, 25–34, normal cytology, in a relationship).

One woman was upset about her result and concerned about her partner's reaction, however he ‘…wasn't interested at all’ and was dismissive of her feelings.

Partners ‘didn't have a clue what it [HPV] was’ and did not understand what the result meant. Knowledge of the HPV vaccine did not necessarily help them understand an HPV‐positive result.

Partners were supportive and understanding following disclosure and concerned about the impact that HPV might have. Others were ‘taken aback’ and ‘overwhelmed’ and felt it was something they needed to get their ‘head ‘round’. Some partners responded defensively, or expressed concerns about transmission of HPV and the impact it might have.

Some partners reacted with humour about the sexually transmitted nature of HPV. One woman who did not view HPV as ‘anything serious’described how she and her partner ‘almost saw it as a bit of a joke’:

‘To be honest, it's not a joke, but we almost took it like that [laughter]. It didn't bother us, [laughs] because it was almost a bit like, “Oh, you've got an STI, ha ha,” it was kind of told like that. We did not, we did not see it as anything serious, erm, because I had not had any symptoms and yeah, it's quite a common thing to get, so it, we almost saw it as a bit of a joke’ (P16, 25–34, normal cytology, in a relationship).

In contrast, another woman was upset by stigmatizing remarks her partner made about her having HPV:

‘And then later, he made this joke, erm ringing a bell and saying “unclean,” and I said to him, “I don't find that funny, I'm really not happy with this. ” And he was like, “Oh okay, sorry,” [laughs] and he actually made it, I told him about this research, and he went, “Oh, the unclean phone‐call,” and rang the little bell again. And I was like, “You're not funny, you're really not funny.”’ (P17, 35–44, normal cytology, in a relationship).

### Feelings about future sexual relationships and disclosure

#### Strategies to prevent transmission

Women, often those not in a relationship, questioned what they could do to prevent HPV transmission and avoid being re‐infected in the future, perhaps by using condoms:

I'm just more concerned about just making sure that we use adequate protection, just for the benefit of both people, you know, myself and the guy I'm with, just to make sure that you know, you don't get anything. Not just HPV, but anything else as well, just you know, just be cautious on that front (P13, 45–54, normal cytology, not in a relationship).

Others felt there was ‘no point’ as their partner had probably already been infected. Receiving an HPV‐positive result was described as ‘a wake‐up call’ and some women felt they would be more likely to use condoms in the future. To avoid transmitting HPV, waiting until their next screening test before having sex to see if their HPV infection had cleared was described:

…I would probably wait until September because, erm, my next smear is in September… And if I get a… like you know, like a negative result then, then I'd be happy to, but until then I don't, I don't feel like I want to, I don't feel like I'd want to at all… Erm, just in case I have still got it and then I pass it on to somebody else (P23, 25–34, abnormal cytology, not in a relationship).

Asking a partner to have a sexual health check before a new sexual relationship was mentioned. For some women this was something they had done prior to having HPV but now felt it was even more important. Questions were raised about HPV testing for men and whether HPV testing was part of a sexual health screening. Women felt that if there was an HPV test for men, they might be able to prevent passing on the infection to women.

#### Feelings about future sexual relationships

Concerns about acquiring another STI led women to feel ‘wary’ or ‘cautious’ about future sexual relationships, particularly when they had acquired HPV despite having had a sexual health screening before starting a new relationship and using protection:

Initially I probably thought oh my gosh, I never wanna have sex again, um, thinking that, you know, if you can catch it when you've used protection and you've been careful about the choice of partners, there was definitely that bit of is it worth it. Is it worth having sex if you can catch these viruses… (P9, 35–44, normal cytology, not in a relationship).

For others, having HPV had not affected their feelings about future relationships or sexual relationships:

I think it is something that I can put to the back of my head. Um, and, you know, I'm not gonna let it stop me but, um, I've just gotta look out for any symptoms, you know. I'm not gonna put my life on hold because of that (P10, 25–34, normal cytology, not in a relationship).

### Factors influencing emotional and psychosocial responses

Several factors appeared to influence women's emotional and psychosocial responses and minimize the negative psychosexual impact of testing HPV‐positive.

#### How women conceptualized HPV

HPV was described as ‘…like an STD’, but also as something that was transmitted ‘…through sexual contact…but not an STI’. There was uncertainty around how HPV should be labelled (i.e. as a virus or STI). The term ‘HPV‐positive’ contributed to women's conceptualization of HPV as an STI because of the similarity to ‘HIV positive’.

Women's certainty about HPV being sexually transmitted varied, with information from healthcare professionals appearing to influence beliefs. The influence other factors might have in causing HPV, such as whether all women have HPV and it is ‘activated’ by stress or hormones or having a ‘low immune system’ was described:

And obviously they're saying it's sexually transmitted but…my understanding was that also it's a bit like a herpes virus in that everybody has it in them but in some people it's activated and in other it lies dormant. Um, so I, the only thing I'm not clear on is if it's something we all have in us that is just activated now and then, by stress or hormones or whatever, or if it is actually only got from sexual transmission (P11, 45–54, normal cytology, not in a relationship).

Understanding the high prevalence and asymptomatic nature of HPV and that it was something that could clear by itself without treatment appeared to minimize its impact. In contrast, a lack of knowledge also appeared to act as a buffer to negative emotional and psychosocial responses. If HPV was not part of sexual health screening then it could not be a ‘serious sexual thing’. Women who did not consider HPV an STI did not feel embarrassed or ashamed disclosing as it was just a ‘biological medical thing’ similar to an abnormal cytology result. A lack of concern about transmitting HPV because it was not ‘…like gonorrhoea or syphilis or HIV or something where you could endanger somebody else’ was mentioned.

I think that the taboo of having an STI can be really embarrassing, err so yeah, I think probably people could be embarrassed by it. Not, not me so much, because I know, I'm in, you know, a long‐term relationship and it's just something I could have picked up… Erm I think a lot of people know what Chlamydia is, well, I didn't know what HPV was, but if someone said Chlamydia, you know what it is, and you associate it with certain… It's wrong, but you stereotype it a little bit. Whereas because I hadn't heard of HPV, to me it wasn't kind of in the same area as Chlamydia (P16, 25–34, normal cytology, in a relationship).

#### HPV dormancy

Women understood that HPV could stay in the body for years or lie dormant which provided reassurance that it had not come from their current partner, reducing concerns about infidelity. However, this also caused confusion. Women questioned how long it could lie dormant for, why it had not ‘shown up’ on previous screening tests and how it could be present when they had not recently been sexually active. Beliefs about dormancy influenced women's beliefs about the source of their HPV infection:

…I think I know that you can carry this virus around yourself for years and years and years and it might not come to anything and then all of a sudden… so it could have been something from years previously, as it most probably was with me (P15, 35–44, normal cytology, not in a relationship).

#### Concern about transmitting HPV

While some women were concerned about transmitting their HPV infection to their partner, others felt that HPV was something that was ‘more harmful to a female than it was to a male’. This belief allowed women to justify why they had not disclosed having HPV to a male sexual partner. Women felt that they would be more concerned about transmitting HPV if they were single, however believing that they had probably already transmitted HPV to their long‐term partner provided reassurance:

I think what got me over it was if I was going to infect him with it, I already have. Erm and it's not something that could hurt him long‐term (P17, 35–44, normal cytology, in a relationship).

#### Persistent HPV infection

Some women who had tested HPV‐positive more than once mentioned that HPV did have an impact on their sexual relationship when they received their first result. However, at the time of the interview it was no longer having an impact, partly because their knowledge about HPV had increased.

I think originally I'd been worried you know, like, who did I get it from or how did I get it? And now that I think like it's – it's quite normal, I don't worry about it as much (P4, 25–34, abnormal cytology, in a relationship).

## DISCUSSION

We explored the psychosexual impact and disclosure experiences of women who had tested HPV‐positive in the context of HPV‐based cervical screening. The sexually transmitted nature of HPV, and concerns about transmission of HPV and where their HPV infection had come from, impacted women's current, past and future interpersonal and sexual relationships. However, psychosexual impact varied. Women's psychosexual response was influenced by how they conceptualized HPV, their understanding of key aspects of HPV such as its high prevalence and dormancy, concerns about transmitting HPV and having a persistent HPV infection.

Responses to testing HPV‐positive such as questioning the source of their HPV infection, concerns about transmitting HPV to a sexual partner, reduced interest in sex and concerns about disclosure because of the stigma that is attached to having an STI are consistent with previous research (Bennett et al., 2019; Bennett, Waller, Ryan, et al., 2021b). Feeling ‘dirty’, embarrassed and ashamed, and concerned about disclosure are common responses among individuals diagnosed with other STIs (Melville et al., 2003; Mortensen & Larsen, 2010; Pavlin et al., 2006). Viewing HPV as different from other STIs appeared to minimize psychosexual impact. HPV differs from other STIs as it is normally asymptomatic and does not require treatment or contact‐tracing. Highlighting these differences may help reduce the stigma and negative psychosexual impact of testing HPV‐positive.

Women's knowledge and conceptualization of HPV influenced responses to testing positive. Previous qualitative research has identified knowledge of HPV's high prevalence, spontaneous clearance, aspects relating to transmission and dormancy and how women conceptualize HPV as factors which can influence and potentially minimize the adverse psychosocial impact of testing HPV‐positive (McCaffery et al., 2006; O'Connor et al., 2014; Waller et al., 2005). These factors may help explain variation in psychosexual response and should be explored in quantitative studies.

Previous research suggests that women who do not have a partner have poorer psychosexual outcomes than women who do (Daley et al., 2015; Hsu et al., 2018). Only women in a relationship mentioned the impact of an HPV‐positive result on sex and sexual relationships (e.g. reduced interest in and frequency of sex) and concerns about trust and infidelity. However, negative emotions, questions about the source of their HPV infection and concerns about transmitting HPV were mentioned by both women in a relationship and not in a relationship. Our findings highlight the importance of ensuring that information about HPV and relationships is available when screening results are provided.

Our findings suggest that a range of factors influence women's decision to disclose, such as feeling that disclosure is the right thing to do and concern about how their partner would respond. Similar factors influence disclosure decisions among participants with other STIs (Keller et al., 2000; Myers et al., 2016). Some women questioned whether it was necessary to disclose HPV to a sexual partner. Since HPV primary screening was rolled out across England in 2019, women testing HPV‐positive receive guidance stating that they do not need to disclose having HPV if they do not want to. It is unclear how many women in this study received this guidance and for some, this may have resolved the questions they had around disclosure. However, some said that they found the guidance confusing and unhelpful. Future research should explore women's understanding of this guidance and whether there are any additional questions about disclosure that should be addressed.

### Strengths and limitations

This study is the first to explore psychosexual and disclosure‐related outcomes among women testing HPV‐positive in‐depth. The sample included women who varied by age, ethnicity, education and those who did/did not feel their HPV‐positive result had any psychosexual impact.

The timing from when women received their screening results to when they were interviewed ranged from a few weeks to nearly a year. In addition, women were recruited based on self‐reported HPV result. It is possible that interviewees were susceptible to recall bias. Data were not collected on sexual orientation or gender identity. Women who have sex with women may have different psychosexual concerns compared to women who have sex exclusively with men. Attending cervical screening can cause gender dysphoria among transgender men and non‐binary individuals and it is possible that psychosexual distress following an HPV‐positive result may be different among these groups compared to cisgender individuals (Connolly et al., 2020). Future research should explore this.

### Practice implications

Increasing knowledge of the key aspects of HPV, such as its high prevalence, that it can clear without treatment in most cases and that there is no need to tell a partner about the infection, may help to mitigate any psychosexual consequences of testing HPV‐positive. Information should be provided in screening materials and results letters for women testing HPV‐positive. Healthcare professionals can play a key role in minimizing any adverse psychosexual consequences of testing HPV‐positive. Healthcare professionals carrying out cervical screening could be trained to give brief information during screening to ensure that women understand their results when they receive them.

In England, most cervical screening is carried out in primary care and it is likely that healthcare professionals working in this setting will be the first point of call for many women testing HPV‐positive who have psychosexual concerns. However, given that HPV is sexually transmitted, healthcare professionals working in sexual health services may also be approached by women.

Previous research with clinicians has identified a number of barriers to discussing an HPV infection with women, many due to HPV's sexually transmitted nature (e.g. embarrassment, not wanting to pass judgement on patients' sexual behaviour, concern that a patient might think they have a sexually transmitted infection or think that their partner is being unfaithful) (McSherry et al., 2012). It is vital that healthcare professionals have adequate knowledge about HPV and feel comfortable and confident responding to women's concerns, which may require additional training.

How women conceptualized HPV influenced psychosexual response. Referring to HPV as an infection that is passed on by skin‐to‐skin contact during sexual activity, rather than an STI, may help to reduce any psychosexual impact triggered by the STI label. This is an approach already being advocated by a UK‐based cervical cancer charity in an attempt to reduce stigma, fear and confusion about HPV (Jo's Cervical Cancer Trust, 2020a; Jo's Cervical Cancer Trust, 2020b).

## CONCLUSION

Testing HPV‐positive can result in adverse emotional and psychosocial responses impacting interpersonal and sexual relationships. However, some women appeared to be relatively unaffected by their HPV‐positive result. Several factors appeared to influence this. The findings suggest that increasing knowledge of key aspects of HPV, such as its high prevalence and spontaneous clearance, and the differences between HPV and other STIs, may increase women's understanding of their screening result and reduce any potential negative psychosexual consequences of testing HPV‐positive.

## AUTHOR CONTRIBUTIONS

**Kirsty F Bennett:** Conceptualization; formal analysis; investigation; methodology; project administration; writing – original draft. **Jo Waller:** Conceptualization; formal analysis; methodology; writing – review and editing. **Julia V Bailey:** Conceptualization; formal analysis; methodology; writing – review and editing. **Laura AV Marlow:** Conceptualization; formal analysis; methodology; writing – review and editing.

## CONFLICT OF INTEREST

All authors declare no conflict of interest.

## Data Availability

Interview transcripts will not be shared to protect participant confidentiality. The summary framework charts that support the findings of this study are available from the corresponding author upon reasonable request.

## Endnote

^1^
Participant ID number, age category (years), cytology result and relationship status.
