# Supplementary material for: Exploring the psychosexual impact and disclosure experiences of women testing positive for high‐risk cervical human papillomavirus
Source: Br J Health Psychol. 2022 Jul 12;28(1):62–79. doi: 10.1111/bjhp.12612 (PMC10084302; doi:10.1111/bjhp.12612)
Supplement: Supplementary file 1 [file BJHP-28-62-s001.pdf]

HPV: impact on sex and relationships

Topic Guide for interviews

1. **Introduction**
   - Thanks for agreeing to take part.
   - Introduce self and UCL – emphasise that we do not work for the screening programme or NHS.
   - Explain what study is about – e.g. screening for HPV is a new way of examining cervical screening (smear test) samples. With the change to cervical screening we'd like to talk to women who have been told that they have HPV to find out how they feel about their screening result, including how this has impacted sex and relationships.
   - No right or wrong answers.
   - Reminder of right to withdraw – confidentiality, anonymity, don't have to answer any questions don't want to.
   - Overview: knowledge and experience of cervical screening and HPV, questions about sex and relationships, information you were given. Length of interview.
   - Audio-recording – double check consent.
   - Any questions before we start?

2. **Knowledge of cervical screening and HPV**

First of all I'm going to ask you some questions about what you know about cervical screening and HPV. Emphasise no right or wrong answers and that it's not a test.

- Can you tell me what you know about cervical screening?
  - Purpose of screening? What is the test looking for?
- Before being told you had HPV had you heard of it before?
- Can you tell me what you know about HPV?
  - How do you get it?
- Where did knowledge come from (e.g. health professionals, screening materials, friends, adverts, online?)?

3. **Experience of cervical screening and testing positive for HPV**

I'm now going to ask you some questions about your experience of cervical screening and being told you have HPV.

- Can you tell me about your experience of being told you had HPV?
  - When were you told you had HPV?
  - How were you told you had HPV (letter/verbally)?
  - Was this the first time you were told you had HPV?
  - Have you had an abnormal cervical screening result before?
  - Do you remember your cytology result (normal: no cell changes found/abnormal: cell changes found)? What do you think the results mean?
  - Initial feelings following screening result?
  - Questions about HPV/screening result?

4. Impact on sex and relationships

I'm now going to ask you some questions about sex and relationships. Remind participants that they don't have to answer questions if they don't want to. For these questions it would be helpful to know whether you currently have a regular sexual partner(s) so I can tailor the questions to you.

- Did you talk to anyone about having HPV? (who?)
    - Reasons for disclosure/non-disclosure?
    - How did you feel about talking to others about having HPV?
    - Reactions to disclosure?

- **If participant <u>does not</u> currently have a regular partner:** intentions to disclose to future sexual partner?

- Has having HPV had any impact on your relationships with others? (partner, friends, family) (could be positive or negative)
    - Changes to relationship(s)?
    - Worries or concerns about relationship(s)?
    - Changes to feelings about partner(s)?
    - If HPV had an impact how long did this last for?

- **If participant <u>does not</u> currently have a regular partner:** feelings about future relationships?

- Has having HPV had any impact on your sex life? (could be positive or negative) – Remind participants that they don't have to answer questions if they don't want to**. Questions can be asked to participants with and without a regular partner.**
    - Changes? (e.g. frequency, interest, satisfaction with sex, practical changes – e.g. changes in condom use?)
    - Worries or concerns about having sex?
    - Feelings about sex? Any change after diagnosis?
    - If HPV had an impact how long did this last for?

5. Communication and information needs

- Do you remember being given any information about HPV (written or verbally)?
- Thoughts about information provided (screening leaflet, invitation and result letters, communication from HCP's)?
- Lacking any information?
- Did you look for information anywhere else (if so where?)?
- Suggestions for improvements/changes to information?

6. Closing the interview

- Thank you
- Ask participant to complete demographics questionnaire.
- Any questions or comments? Anything important to you that you haven't already mentioned? Extra comments about HPV or cervical screening in general?
- Reassure about confidentiality
- What happens next
